# Spermidine dietary supplementation and polyamines level in reference to survival and lifespan of honey bees

**DOI:** 10.1038/s41598-023-31456-4

**Published:** 2023-03-15

**Authors:** Srđana Đorđievski, Elvira L. Vukašinović, Tatjana V. Čelić, Ivan Pihler, Marko Kebert, Danijela Kojić, Jelena Purać

**Affiliations:** 1 Department of Biology and Ecology, Faculty of Sciences, University of Novi Sad, Novi Sad, Serbia; 2 Departmnent of Animal Sciences, Faculty of Agriculture, University of Novi Sad, Novi Sad, Serbia; 3 Institute of Lowland Forestry and Environment, University of Novi Sad, Novi Sad, Serbia

**Keywords:** Biochemistry, Molecular biology

## Abstract

Honey bee health has been an important and ongoing topic in recent years. Honey bee is also an important model organism for aging studies. Polyamines, putrescine, spermidine and spermine, are ubiquitous polycations, involved in a wide range of cellular processes such as cell growth, gene regulation, immunity, and regulation of lifespan. Spermidine, named longevity elixir, has been most analysed in the context of aging. One of the several proposed mechanisms behind spermidine actions is antioxidative activity. In present study we showed that dietary spermidine supplementation: (a) improved survival, (b) increased the average lifespan, (c) influenced the content of endogenous polyamines by increasing the level of putrescine and spermidine and decreasing the level of spermine, (d) reduced oxidative stress (MDA level), (e) increased the antioxidant capacity of the organism (FRAP), (f) increased relative gene expression of five genes involved in polyamine metabolism, and (g) upregulated vitellogenin gene in honey bees. To our knowledge, this is the first study on honey bee polyamine levels in reference to their longevity. These results provide important information on possible strategies for improving honey bee health by introducing spermidine into their diet. Here, we offer spermidine concentrations that could be considered for that purpose.

## Introduction

**Figure 1:**
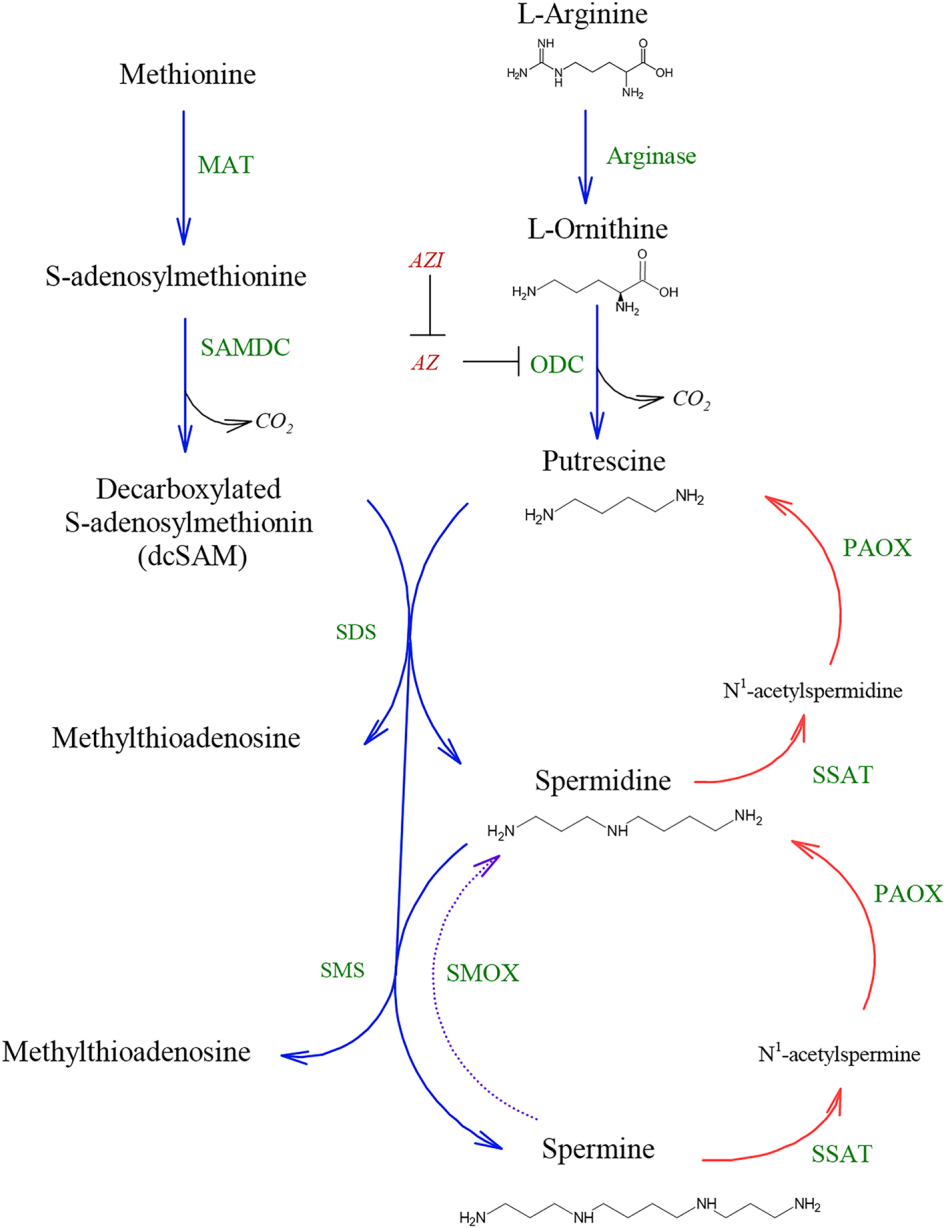
Polyamines metabolic pathway. Ornithine decarboxylase (ODC); spermidine synthase (SDS); spermine synthase (SMS); methyl adenosyl transferase (MAT); S-adenosyl methionine decarboxylase (SAMDC); spermine oxidase (SMOX); spermidine/spermine N1-acetyltrasferase (SSAT); polyamine oxidase (PAOX); antizyme (AZ); antizyme inhibitor (AZI).

Polyamines are positively charged molecules present in all living organisms, where they interact with negatively charged molecules such as DNA, RNA, ATP, proteins, and phospholipids^1^, involved in various cellular processes including cell growth, gene regulation, differentiation, development, and immunity^2^. The most abundant polyamines are diamine putrescine, polyamines spermidine, and spermine^1^, which are part of tightly regulated polyamines metabolic pathway^3^. Polyamines content in cell is regulated by biosynthesis, degradation, uptake, and excretion^3,4^. Figure 1 represent a metabolic pathway of polyamines in eukaryotic cell, for more details see^2,5^

One of the most studied polyamines is spermidine. In the recent studies focused on mechanisms of aging, spermidine has emerged as a molecule with anti-aging properties^1,6,7^. Although spermidine concentration in tissue declines with age^8^, its oral supplementation through food or water increases spermidine levels in both model organisms and humans^9^. This provides an ideal opportunity to investigate the effects of spermidine on age and longevity. Moreover, available evidence indicates that external spermidine supplementation prolongs lifespan and healthspan in a variety of species and cultured human immune cells^6^. Additionally, spermidine increases resistance to stress in yeast and flies, inhibits oxidative stress in aging mice and shows health promoting effects in humans^1,6,10–12^. Spermidine exhibits anti-inflammatory and antioxidant properties, enhances mitochondrial metabolic function and respiration, promotes chaperone activity, and improves proteostasis^1,12^. The mechanisms behind the broad effect of spermidine action have not yet been fully elucidated. Induction of autophagy is considered as the main mechanism of spermidine action. However, there are other mechanisms for which it has not been formally determined whether they act in a completely autophagy-independent manner, including the antioxidant effect of this molecule^1,10^.

Honey bee (*Apis mellifera* L.), as a prominent group of pollinator species worldwide, is an important component of global biodiversity, providing important ecosystem service in the form of crop and wild plant pollination^13^. A significant decline in the managed honey bee populations both in Europe and the USA has been documented in numerous studies over the past decades^13,14^. Many researchers support the view that this decline can be attributed to multiple harmful factors that cause chronic sublethal stress, such as pesticide use, malnutrition, habitat loss, environmental conditions, parasites, and pathogens, as well as poor management practices^15,16^. Due to these adverse trends, the health and survival of the honey bees has been the subject of growing scientific interest in recent years.

Honey bee is recognized as a model for studying the biology of aging^17,18^, because of highly pronounced aging plasticity. All bees in one hive could have similar genotype; however, summer workers usually live up to six weeks, whereas winter workers live for six months or more, whereby this difference in lifespan is mostly regulated by environmental factors^19^. Bees are therefore an excellent model for testing the effects of individual molecules on aging processes and lifespan regulation. One of the best-studied candidates for regulation of the aging plasticity in honey bees is vitellogenin. This phosphoglycolipo-protein is a female-specific egg yolk precursor that is common to most oviparous animals, however, in bees it was also found in sterile workers, implying additional roles. Vitellogenin is an important regulator of aging, with well-documented influence on behaviour, oxidative stress resistance, cell-based immunity, and longevity in honey bees^20–22^.

According to our knowledge, no studies exploring spermidine effects on honey bee health and longevity have been published to date. The aim of this study was to examine the influence of dietary spermidine supplementation on survival rate, average lifespan, endogenous polyamines content, oxidative stress and relative expression of vitellogenin gene and selected genes involved in polyamines metabolism in honey bees. These results will be supported by information on the influence of aging on endogenous polyamine content and oxidative stress parameters. Obtained results will be interesting for at least two reasons. The first is that if a positive effect of spermidine on health and longevity is shown, further research can be carried out with the aim of better elucidating the mechanism of its action and spermidine could have a potential practical application in beekeeping. The second reason is the fact that this type of studies on honey bees, as important aging model, could improve our understanding of molecular mechanisms underlying aging in general.

## Results

### Survival and average lifespan

**Figure 2:**
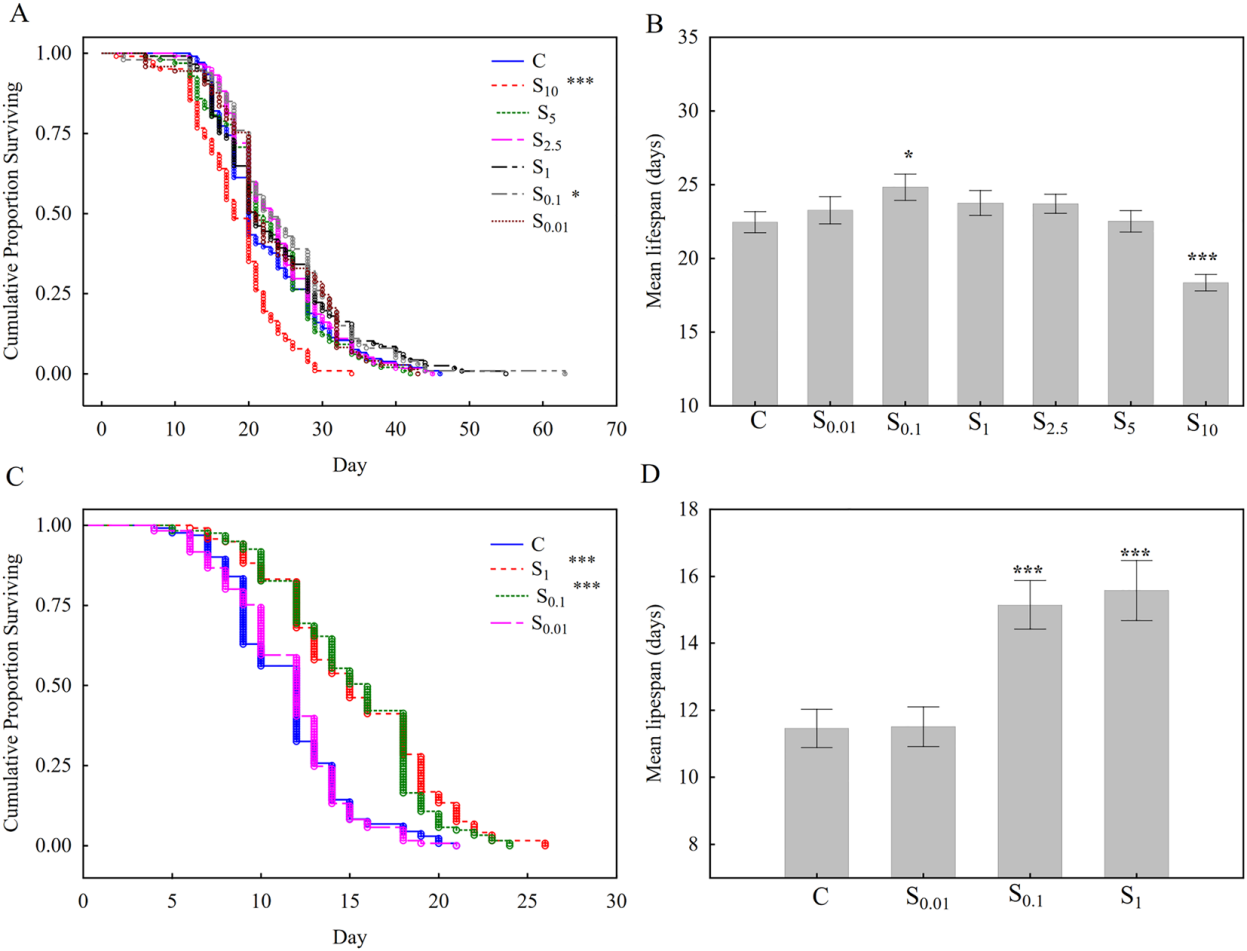
Cumulative Proportion Surviving (Kaplan–Meier) throughout 63 days (**A**) and 34 days (**C**) for honey bees. Average lifespan in analysed experimental groups (**B**) (**D**). Control group C was fed with 50% sucrose solution. Experimental groups S_0.01_, S_0.1_, S_1_, S_2.5_, S_5_ and S_10_ were fed with sucrose supplemented with spermidine concentrations: 0.01, 0.1, 1, 2.5, 5 and 10 mM, respectively. Sample size for (**A**) and (**B**): C (n = 108), S_0.01_ (n = 73), S_0.1_ (n = 99), S_1_ (n = 117), S_2.5_ (n = 118), S_5_ (n = 99), S_10_ (n = 103); sample size for (**C**) and (**D**): C (n = 132), S_0.01_ (n = 123), S_0.1_ (n = 121), S_1_ (n = 120). Kaplan–Meier curves were analysed with log rank test and statistically significant difference compered to control is shown. Average lifespan is shown in days with standard error (SE); the differences between average values of each spermidine treatment and control were analysed by t-test (*p < 0.05, ***p < 0.001).

In the first experiment (spermidine concentrations 0.01, 0.1, 1, 2.5, 5, and 10 mM) the results showed that the diet supplemented with 0.1 mM spermidine increased, while the diet supplemented with 10 mM spermidine decreased cumulative survival rate (Fig. 2A) and average lifespan (Fig. 2B). Spermidine concentrations 0.01 and 1 mM also showed positive effect on survival rate, but without statistical significance, therefore experiment was repeated one more time with these experimental groups. In the repeated experiment (spermidine concentrations 0.01, 0.1, and 1 mM) obtained results showed that S_0.1_ group, S_1_ groups significantely increased survival rate (Fig. 2C) and average lifespan (Fig. 2D) of honey bees compared to control, so these two concentrations were selected for further analysis.

It is necessary to note that the maximum and average length of life were much longer in the first experiment than in the second. Possible reasons might be different time of the year when the frames with broods were taken (environmental factors) and/or different genetics, as indicated in many studies^23–25^. However the focus of these experiments was the effect of spermidine on lifespan, not their maximum length of life, and in that sense the key fact for comparing their lifespan was that all bees (supplemented and control) were under the same conditions.

### Spermidine intake and honey bee mortality

**Figure 3:**
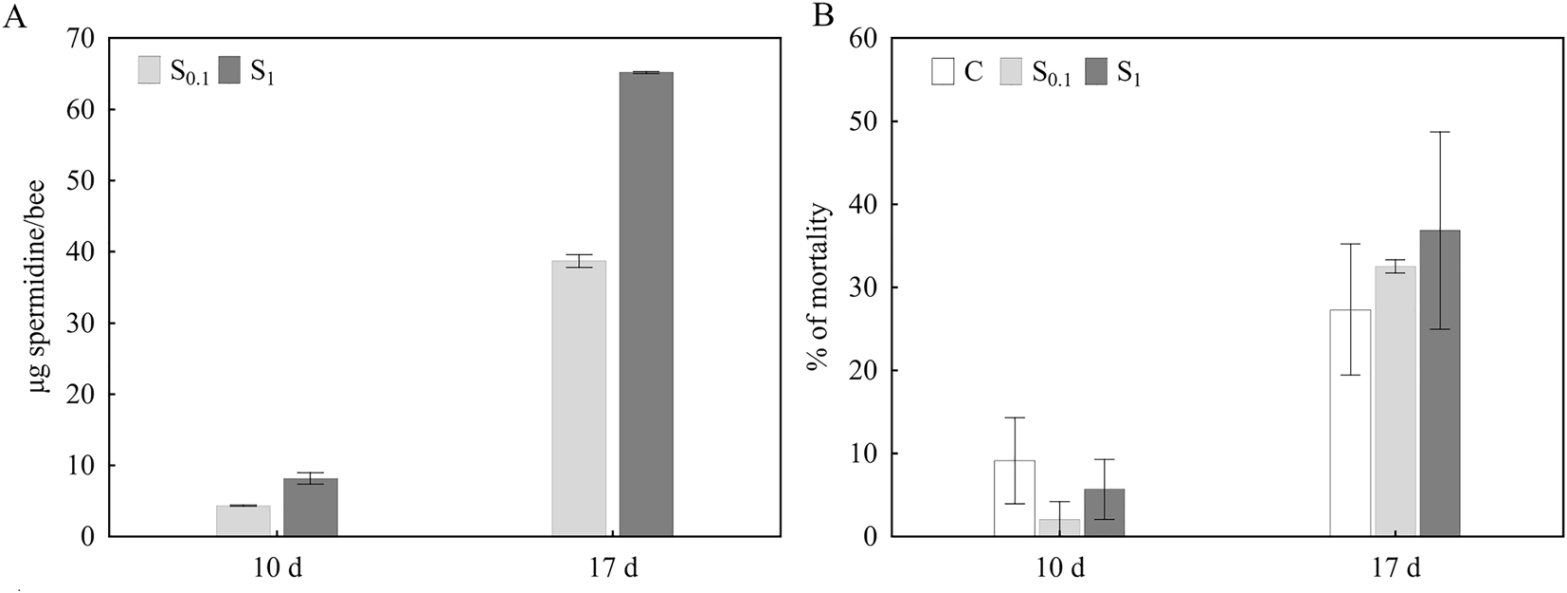
(**A**) Total mass of spermidine in µg which was ingested in 10 (10d) or 17 days (17d) per bee. (**B**) Mortality in analyzed experimental groups after 10 days (10d) and 17 days (17d). Control group C was fed with 50% sucrose solution, while in S_0.1_ and S_1_ groups diet was supplemented with 0.1 mM and 1 mM spermidine, respectively. Sample size: C 10d (n = 100), C 17d (n = 90), S_0.1_ 10d (n = 96), S_0.1_ 17d (n = 80), S_1_ 10d (n = 102), S_1_ 17d (n = 87). Mean value ± standard error (SE) is presented.

The mass of ingested spermidine for 10 and 17 days, respectively, was calculated based on the total volume that was eaten on average by one bee in each time period, in each experimental group. After 10 days, 4.29 µg spermidine in the S_0.1_ group and 38.68 µg spermidine in the S1 group was ingested per bee. After 17 days, 8.14 µg of spermidine in the S_0.1_ group and 65.21 µg of spermidine in the S_1_ group was ingested per bee. (Fig. 3A). Mortality after 10 days was: 9.1% in the control, 2.1% in the S_0.1_ and 5.7% in the S_1_ group. Mortality after 17 days was: 27.3% in the control, 32.5% in the S_0.1_ and 36.9% in the S_1_ group (Fig. 3B). There were no statistically significant differences in mortality between different experimental groups after 10 days or after 17 days.

### HPLC analysis of polyamines

**Figure 4:**
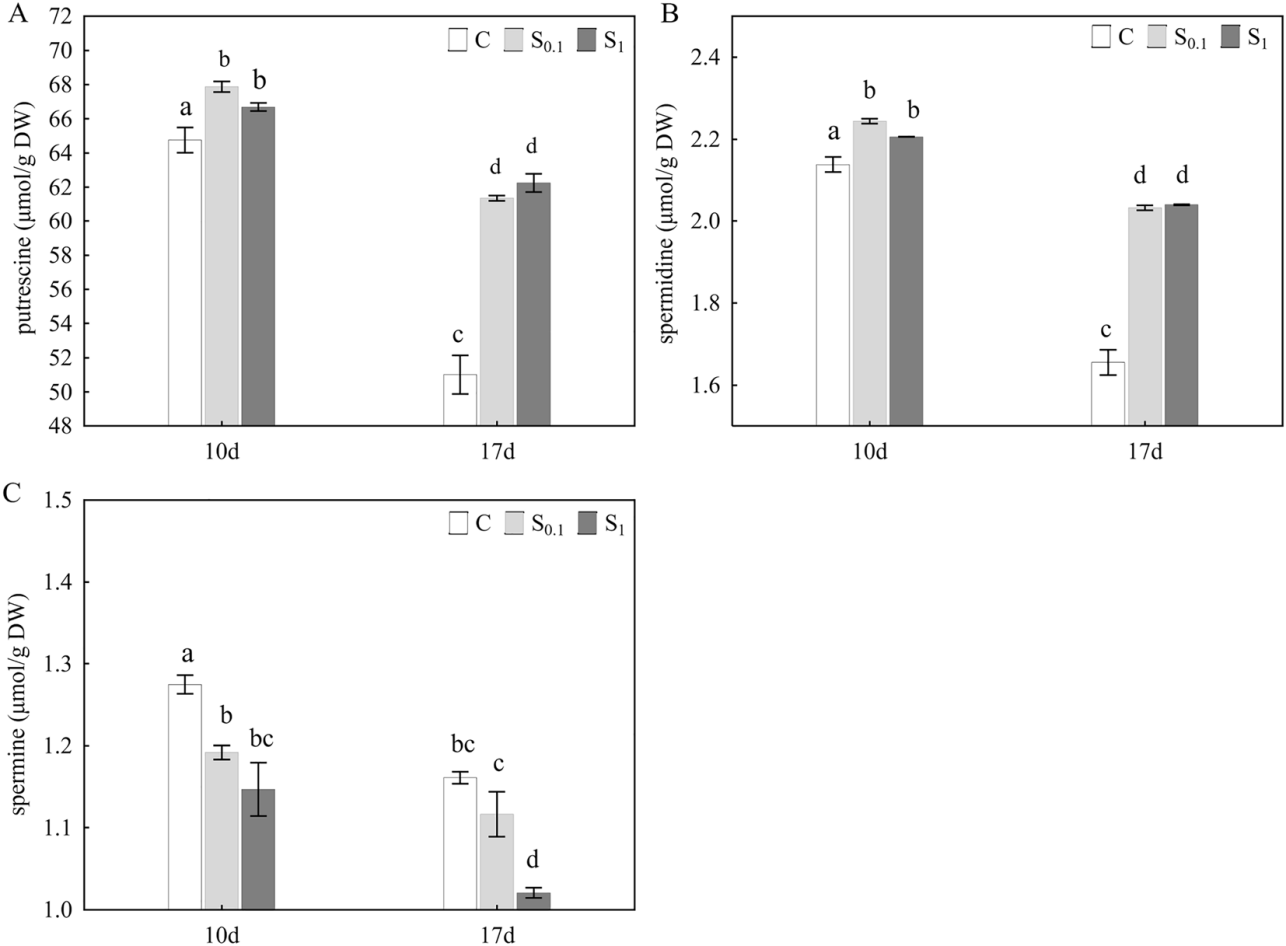
Putrescine (**A**), spermidine (**B**) and spermine (**C**) concentration in honey bee body in µmol/g DW (dry weight) after 10 (10d) and 17 days (17d). Control C was fed with 50% sucrose solution, and in groups S_0.1_ and S_1_ diet was supplemented with 0.1 mM and 1 mM spermidine, respectively. Data is presented as mean ± standard error (SE). Differences between mean values were analyzed with one-way ANOVA and post-hoc Duncan’s test. Different letters indicate differences among all experimental groups shown on the graph for *p < 0.05.

The content of biogenic polyamines, analysed by HPLC, is shown in Fig. 4. From comparison between the control groups after 10 and 17 days, it is clear that the concentration of all three polyamines (Fig. 4A, B, C) decreased with the aging of honey bees. Dietary spermidine supplementation affected putrescine and spermidine content in the same way, by increasing their concentration in S_0.1_ and S_1_ groups compared to control, after 10 and 17 days (Fig. 4A, B). Interestingly, there was no difference in the concentration of putrescine (Fig. 4A) and spermidine (Fig. 4B) between the S_0.1_ and S_1_ groups either after 10 or after 17 days, even though the bees in the S_1_ group were fed a tenfold higher concentration of spermidine. In contrast, spermine concentration was significantly decreased in honey bees fed with 0.1 mM and 1 mM spermidine, after 10 and 17 days, compared to control (Fig. 4C).

### MDA and FRAP

**Figure 5:**
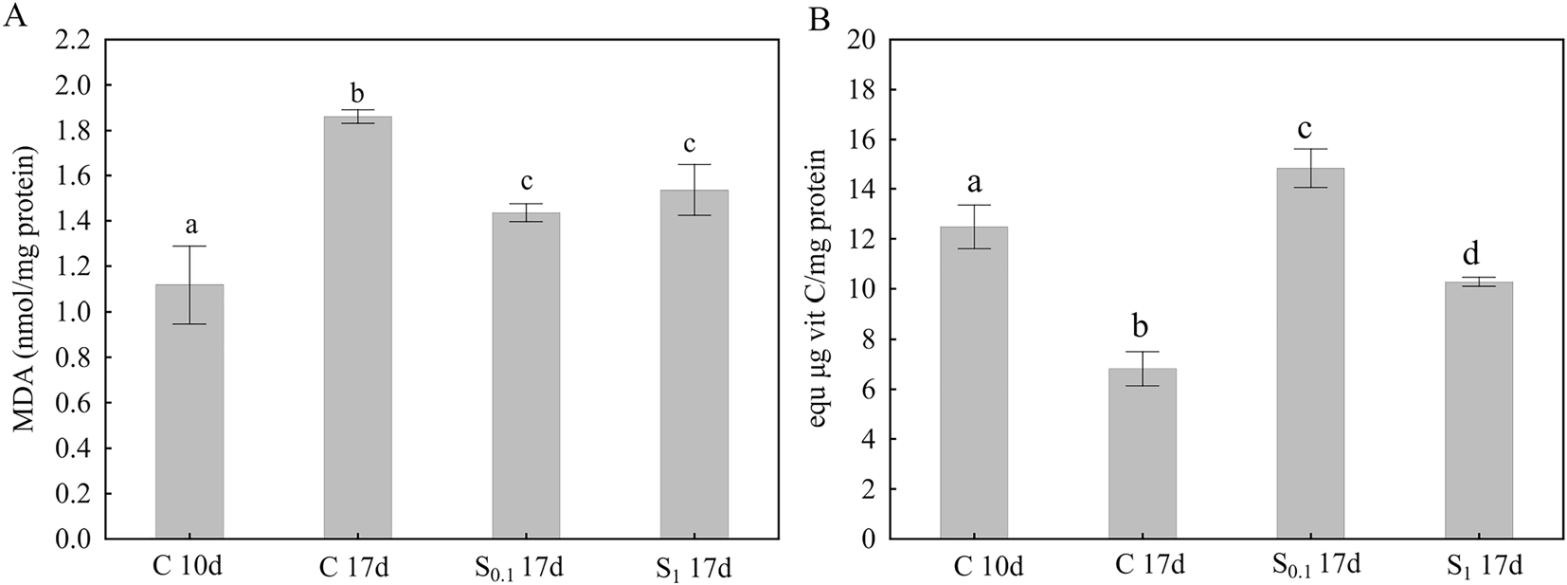
MDA level (nmol/mg) (**A**) and FRAP (equ µg vit C/mg protein) (**B**) after 10 (10d) and 17 days (17d) in honey bee. Control C was fed with 50% sucrose solution, and in groups S_0.1_ and S_1_ diet was supplemented with 0.1 mM and 1 mM spermidine, respectively. Data is presented as mean ± standard error (SE). Differences between mean values were analyzed with one-way ANOVA and post-hoc Duncan’s test. Different letters indicate differences among all experimental groups shown on the graph for *p* < 0.05.

The results of MDA and FRAP analysis are shown in Fig. 5. Malondialdehyde (MDA) is a product of lipid peroxidation and has been used as a biomarker of oxidative stress^26,27^. The ferric reducing antioxidant power (FRAP) assay is simple, quick, and inexpensive method of measuring antioxidant activity of reductive antioxidants in sample^28^. MDA was significantly increased in the control group after 17 days when compared to 10 days, which means that MDA increases with age. Supplementation of honey bee diet for 17 days with both concentrations of spermidine 0.1 mM and 1 mM reduced the MDA levels (Fig. 5A). The antioxidant capacity of whole body measured by FRAP assay showed the opposite trend. It decreased with aging, however dietary supplementation with spermidine concentrations 0.1 mM and 1 mM increased antioxidant capacity (Fig. 5B).

### Relative gene expression

Exogenously added spermidine to food in both concentrations 0.1 mM and 1 mM upregulated all analyzed genes (*ODC, SDS, SMS, SMOX, PAOX, Vg*) in abdomen after 17 days of supplementations, with the exception that 1 mM spermidine did not affect *Vg* gene expression (Table 1). In head, 0.1 mM spermidine upregulated all analyzed genes except *SMOX*, while 1 mM spermidine upregulated all except *SMOX* and *Vg* genes (Table 1).

**Table 1:** Relative expression and standard error (SE) for genes ornithine decarboxylase (*ODC*), spermidine synthase (*SDS*), spermine synthase (*SMS*), spermine oxidase (*SMOX*), polyamine oxidase (*PAOX*) and vitellogenin (*Vg*) in head and abdomen of bees whose diet was supplemented with 0.1 mM and 1 mM spermidine for 17 days compared to control, fed only sucrose.

		0.1 mM spermidine				1 mM spermidine			
	Gene	Relative expression		SE	*p* value	Relative expression		SE	*p* value
Head	*PAOX*	2.640	**↑**	1.987–3.519	0.000	1.796	**↑**	1.373–2.316	0.027
	*SDS*	2.120	**↑**	1.495–2.873	0.000	1.860	**↑**	1.433–2.616	0.041
	*SMOX*	2.038	**–**	1.059–3.787	0.065	1.268	–	0.818–1.733	0.299
	*ODC*	2.566	**↑**	1.734–3.683	0.000	1.901	**↑**	1.330–2.569	0.043
	*SMS*	2.042	**↑**	1.453–3.001	0.000	1.532	**↑**	1.053–2.309	0.043
	*Vg*	4.953	**↑**	3.824–6.604	0.000	1.232	–	0.962–1.536	0.236
Abdomen	*PAOX*	5.535	**↑**	4.607–7.173	0.022	3.931	**↑**	2.804–5.511	0.009
	*SDS*	4.980	**↑**	3.536–6.956	0.025	1.888	**↑**	1.450–2.450	0.014
	*SMOX*	4.441	**↑**	3.074–5.596	0.000	2.031	**↑**	1.619–2.445	0.000
	*ODC*	2.517	**↑**	1.891–3.279	0.000	1.578	**↑**	1.274–1.847	0.002
	*SMS*	15.428	**↑**	10.394–26.003	0.015	7.793	**↑**	5.164–12.579	0.017
	*Vg*	5.108	**↑**	2.464–9.397	0.000	0.738	–	0.608–0.952	0.089

The difference in the analyzed gene expression was calculated by REST 2009 and tested for statistical significance by the integrated Bootstrap randomization test between the control and target groups. The results of expression of the investigated transcripts for each sample were tested for significance by a Two-sided Pair Wise Fixed Reallocation Randomisation Test^©^. Statistically significant difference compared to the control group is indicated with an arrow **↑** (*p* < 0.05).

## Discussion

In this study we examine the impact of oral spermidine supplementation on survival rate, average lifespan, endogenous polyamines content (putrescine, spermidine and spermine), oxidative stress (MDA and FRAP), and relative expression of vitellogenin gene and selected genes involved in polyamines metabolism in honey bees. In addition, we analysed the influence of aging on endogenous polyamines content and oxidative stress. Polyamine loss has been repeatedly linked to the aging process; however, this connection has not been investigated in honey bee workers. In present study, the results showed that the diet supplemented with 0.1 mM and 1 mM spermidine increased, while the diet supplemented with 10 mM spermidine decreased cumulative survival rate and average lifespan of bees, compared to the control group fed with sucrose solution. This finding is consistent with previous research in different model organisms. Evidence points to an important role of polyamines in determining longevity. Oral supplementation of spermidine, in similar concentrations as in present study, markedly extended the lifespan of yeast, flies, worms, mice and human immune cells^6,10,29^. Further, life-time consumption of chow containing synthetic polyamines (0.374 mM spermine and 1.54 mM spermidine) increased mouse lifespan^30^. Spermidine concentrations 0.01 mM, 2.5 mM and 5 mM had no significant effect in our study, while in contrast, the highest concentration of spermidine (10 mM) showed toxic effect. That is also in accordance with previous studies proving that higher concentrations of spermidine lead to massive production of reactive oxygen species (ROS), which is the main cause of spermidine toxicity in *E. coli* ^31^.

The endogenous content of polyamines was measured by the HPLC method, and in addition, the relative expression of selected genes involved in polyamine metabolism: *ODC*, *SDS*, *SMS*, *SMOX*, and *PAOX* was monitored. The levels of polyamines in honey bees were in the order putrescine > spermidine > spermine with putrescine being present in about 30 times higher concentration than spermidine and spermine. This is different compared to the levels of polyamines in many mammalian tissues in which spermidine is the most and putrescine the least abundant with the ratios between them easily varying from 2 to 20^7^. Further, our results obtained by comparing the control groups that were fed only sucrose solution after 10 and after 17 days, showed that the content of putrescine, spermidine and spermine decreased with age. These results are in correlation with literature data which shows that despite highly diverse polyamine levels in individual organisms, one commonality is that tissue concentrations of spermidine decline in an age-dependent manner in both model organisms and humans^1,6,8,9,32^.

Further, we showed that dietary supplementation with spermidine at both concentrations 0.1 mM and 1 mM changed the content of endogenous polyamines by increasing the level of putrescine and spermidine and on the other hand decreasing spermine concentration, after both 10 and 17 days of supplementation. It is interesting to observe that in the bees who ingested different amounts of spermidine, a similar increase in putrescine and spermidine was recorded both after 10 and 17 days. It is possible that there is an upper physiological limit in polyamine concentration characteristic for age. The relative expression of all analyzed genes involved in polyamines metabolism was increased after 17 days in both abdomen and head when diet was supplemented with 0.1 mM and 1 mM spermidine, except *SMOX* gene in head whose expression didn’t change. Increased expression of genes whose products are involved in the synthesis of putrescine (*ODC*), spermidine (*SDS*) and spermine (*SMS*) as well as in the back conversion of spermine to spermidine (*SMOX*, *PAOX*) or spermidine to putrescine (*PAOX*) indicates an intensified metabolism of polyamines, except for the spermidine synthesis pathway from spermine via the SMOX enzyme in the head. Our results related to the influence of exogenous spermidine on the increase of endogenous spermidine levels are consistent with literature data. Exogenous spermidine in food or water increased its endogenous levels in yeast, flies, mice and humans^6,29^ However reduced level of spermine caused by the spermidine supplemented diet, as shown in this study, indicate complexity of polyamine metabolism and potential inhibitor effect of some molecules. It is known that different polyamines are of different importance to different organisms where the role of spermine in cell functioning is insignificant in lower organisms and important in highly developed animals^33^. Our results, which showed that spermine is the least abundant polyamine in honey bees which decreases after dietary spermidine supplementation, could support this observation. To draw conclusions without speculations, additional research is necessary.

The free radical theory is the most prominent of the theories underlying the aging process. According to this theory, oxidative changes in biomolecules accumulate in the body as an organism age due to the generation of free radicals throughout metabolic pathways^34^. In the present study, we measured MDA level and performed FRAP assay to analyze the occurrence of oxidative stress and capacity for its removal. The results, obtained by comparing the control groups that were fed only sucrose solution after 10 and after 17 days, showed that MDA level increased, while antioxidant capacity decreased with age, which confirms the theory that oxidative stress increases with organism aging^34,35^. Exogenously added spermidine at concentrations 0.1 mM and 1 mM reduced oxidative stress and increased antioxidant capacity after 17 days of supplementation. These results point to antioxidative action of spermidine, which is in accordance with previous studies in mice, yeast, and *D. melanogaster*^6,10,36,37^. Another important regulator of aging in honey bees is vitellogenin. Amdam and Omholt (2002)^38^ proposed a potential role for vitellogenin in honey bee survival. Nelson et al. (2007)^39^ demonstrated that silencing the vitellogenin gene reduces worker bee longevity and lifespan. Our results support these data. In our study oral supplementation with 0.1 mM spermidine upregulated *Vg* gene in both abdomen and head, while 1 mM spermidine didn’t change *Vg* expression. There is not much data in the literature linking biogenic polyamines and vitellogenin. Results obtained by Kogan and Hagedorn (2000)^40^ suggest that polyamines are important for vitellogenesis in the mosquito, which was confirmed by our results (i.e., 0.1 mM spermidine).

To our knowledge, this is the first study on honey bee polyamines level in reference to their longevity. We showed that dietary spermidine supplementation: (a) improved survival, (b) increased the average lifespan, (c) influenced the content of endogenous polyamines by increasing the level of putrescine and spermidine and decreasing the level of spermine, (d) reduced oxidative stress, (e) increased the antioxidant capacity of the organism, (f) increased relative gene expression of five genes involved in polyamine metabolism and (g) upregulated vitellogenin gene. These results provide important information on possible strategies for improving honey bee health by introducing spermidine into their diet. A future perspective may be the development of nutritional supplements that would improve the health and longevity of bees and provide more successful overwintering as one of the biggest problems in beekeeping today. Here, we offer spermidine concentrations that could be considered for that purpose.

## Material and methods

### Experimental setup for analyzing survival and average lifespan

Honey bee workers originated from experimental hives located at the Fruška Gora mountain (45°22' N; 19°53' E), close to Novi Sad, Serbia. The hives were inspected to determine the health status of the worker bees. Colonies were not treated with any chemicals for at least 10 months before the start of the experiment. Frames with brood were taken three times during the spring/summer 2022. For each experiment, two frames with a brood were transferred from hive to the smaller glass observation hive (Supplement Fig. 1A), which was kept in incubator, in the dark at 34 °C and 65% relative humidity to mimic the hive conditions, for the worker bees to hatch (Supplement Fig. 1B). After 24 h, the brood frames were replaced with honey and bee bread frames to provide food for the young bees, and the temperature was lowered to 28 °C. After 5 days, the individuals (between 30 and 55) were randomly transferred to experimental plastic boxes with a volume of 2 L. Smooth aeration was provided by small holes in the box and the bees were fed using a plastic syringe that was pulled inside the box and fixed with adhesive tape (Supplement Fig. 2).

Experiment was repeated two times. First time, the influence of a wide range of spermidine concentrations on survival and lifespan was investigated. Seven experimental groups were formed: C—control, fed with 50% (w/v) sucrose as basic feeding solution, S0.01, S0.1, S1, S2.5, S5, and S10 named after spermidine concentration (mM) in sucrose solution, respectively. The total number of bees per group was as follows: 108 bees in C group, 73 bees in S0.01 group, 99 in S0.1, 117 in S1, 118 in S2.5, 99 in S5 and 103 bees in S10 group. Feeding solutions were freshly prepared every day, from 1 M stock, because of spermidine volatility, and the mass of eaten food was measured. Dead bees were removed every day and their number was recorded. The experiment lasted for 63 days, until the last bee died. Second time, experimental setup was repeated with concentrations that showed positive effect on survival and lifespan. Four experimental groups were formed: control C, fed with 50% sucrose solution, and S0.01, S0.1 and S1 fed with 0.01 mM, 0.1 mM, and 1 mM spermidine, respectively. The total number of bees per group was as follows: 132 bees in C group, 123 bees in S0.01, 121 in S0.1 and 120 in S1 group. The experiment lasted for 34 days, until the last bee died.

### Experimental setup and sampling regime for molecular analysis

The experimental setup for collecting samples for molecular analysis was the same as described above, worker bees were approximately the same age and kept under identical conditions. The difference was that honey bees were collected from plastic boxes after 10 days and after 17 days (10d and 17d, respectively). Bees were fed with sucrose solution in the control group (C) and sucrose solution supplemented with 0.1 mM (S0.1) and 1 mM spermidine (S1) (Table 2), as it was shown for these concentrations to have statistically significant positive effect on survival and lifespan. The total number of bees within each group is given in Table 2. Every day fresh food was prepared, mass of food they ate was measured, dead bees were removed, and their number was recorded. After 10, and after 17 days bees were instantly frozen with dry ice and stored in freezer at -70 °C for further analysis. Biological replicates were formed from these bees. The goal of this experimental setup was to monitor the effect of spermidine supplementation by HPLC, MDA, FRAP and qPCR analysis (comparing supplemented with control groups) and to monitor the effect of aging by HPLC, MDA and FRAP analysis (comparing control groups after 10 and 17 days).

**Table 2:** Overview of experimental groups formed for molecular analysis with total number of bees per group; C-control, fed with 50% (w/v) sucrose as basic feeding solution, in S0.1 and S1 groups food was supplemented with 0.1 and 1 mM spermidine; treatment lasted for 10 and 17 days (10d and 17 d, respectively).

Experimental group	C		S_0.1_		S_1_	
Lenght of treatment	10d	17d	10d	17d	10d	17d
Total number of bees	100	90	96	80	102	87

### HPLC analysis of polyamines

HPLC determination of putrescine, spermidine and spermine content was measured in whole body of honey bees from C, S0.1 and S1 groups, after 10 days and 17 days of experiment. Each HPLC analysis was performed on a pool of two bees, in technical triplicate. Honey bees (approx. 100 mg DW) were lyophilized for 24 h and polyamines were extracted with 2 ml 4% perchloric acid as described by Kebert et. al. (2017)^41^. Homogenates were kept on ice for 1 h and centrifuged at 15 000 × g for 30 min. Supernatants of homogenates and polyamine standards (putrescine, spermidine and spermine) were derivatized with dansyl chloride, extracted with toluen, dried, resuspended with acetonitrile and quantified with HPLC using a reverse phase C18 column (Spherisorb ODS2, 5-lm particle diameter, 4.6 9 250 mm, Waters, Wexford, Ireland), and a programmed acetonitrile–water step gradient. Eluted peaks were detected by a spectrofluorometer (ex. 365 nm, em. 510 nm); the internal standard used was heptamethylenediamine.

### MDA and FRAP analysis

Malondialdehyde (MDA) level and Ferric Reducing Antioxidant Power (FRAP) were measured in whole body of honey bees from control group (C) after 10 days, and from C, S0.1 and S1 groups after 17 days of the experiment. Each experimental group had three biological replicates. Each measurement was performed on a pool of five bees, MDA in technical duplicates and FRAP in technical triplicates. Honey bees were ground to a fine powder in liquid nitrogen with a mortar and pestle and homogenized with ice cold 50 mM phosphate buffer pH 7.0 (20%, w/v). The crude homogenates were centrifuged at 10 000 g (4 °C) for 10 min. The supernatants were used for MDA and FRAP determination. MDA levels were measured according to the method of Rehncorna et al. (1980)^42^, which is based on formation of colored complex (maximum absorption at 532 nm) as a result of reaction of malondialdehyde, the specific product of lipid peroxidation, and thiobarbituric acid (TBA). MDA levels were expressed in nmol per milligram of protein. The antioxidant capacity of whole body was measured by FRAP assay according to the method described by Benzie and Strain (1996)^43^. The method is based on reduction of a colorless ferric-tripyridyltriazine complex (Fe^3+^-TPTZ) into intense blue ferrous-tripyridyltriazine complex (Fe^2+^-TPTZ) in interaction with a potential antioxidant at low pH, with an absorption maximum at 593 nm. Ascorbic acid was used as standard, and results were expressed as equivalent of μg vitamin C per mg of protein. The protein concentration in homogenates was determined using the Bradford method, with bovine γ-globulin as protein standard (Bradford, 1976)^44^.

### Total RNA isolation and cDNA synthesis

Total RNA was extracted from head and abdomen of honey bees from C, S0.1 and S1 groups, after 17 days of the experiment. Each extraction was done from a pool of three bees (three heads, three abdomens) from two biological replicates. Total RNA was extracted using RNA Extracol (EurX) according to manufacturer's protocol and diluted in 30–50 μl RNase/DNase-free water (Sigma-Aldrich). RNA concentration was measured at 260 nm using BioSpec-nano spectrophotometer (Shimatzu), and purity of total RNA was determined as the 260/280 absorbance ratio. After the estimation of purity and concentration, total RNA was diluted to 500 ng/μl and stored at − 70 °C. Synthesis of cDNA was carried out using the Takarra PrimeScript RT Reagent Kit according to manufacturer’s protocol, starting with 1 μg of total RNA and obtained samples were stored at − 20 °C.

### Quantitative PCR

Relative expression was measured for ornithine decarboxylase (ODC), spermidine synthase (SDS), spermine synthase (SMS), spermine oxidase (SMOX), polyamine oxidase (PAOX) and vitellogenin (Vg) genes using gene for ribosomal protein 49 (Rp49) as endogenous control. The suitability of this gene as an endogenous control in qPCR assays, has previously been confirmed^45^. Primers were designed using the NCBI PrimerBlast^46^. Primer efficacy was calculated based on a standard curve with a tenfold dilution series of cDNA. PCR primer sequences and efficiencies are shown in Supplement Table 1. Reaction included 7 μl of 2 × SYBR Green PCR Master Mix (Applied Biosystems), 500 nM of each primer, and 50 ng cDNA in total volume of 14 μl. Quantitative PCR on the cDNA products was carried out using MasterCycler RealPlex4 (Eppendorf). All analyses were performed in technical duplicates. Amplification program consisted of an initial preincubation step at 95 °C (10 min) and 40 cycles of 95 °C (15 s) and 60 °C (1 min) with additional step for melting curve analysis to confirm amplification of a single gene product.

### Data analysis

Statistical data processing was done in the program Statistica v13. Survival curves were created using the method of Kaplan and Meier. The log-rank test was used to evaluate differences between survivals and determine p values. The statistical significance of the results was evaluated with the independent t-test when only two means were compared (lifespan analysis) or with one-way ANOVA followed by Duncan's multiple comparisons test otherwise (mortality, polyamines content, MDA level and FRAP). Differences in expression between control and treated samples were assessed for statistical significance by the integrated randomization and bootstrapping methods within REST 2009 software (Qiagen). REST software utilises non-parametric bootstrapping techniques to calculate the appropriate errors and probability (p) value for the comparison between groups. Software performs up to 10,000 (in our analysis 2000) random reallocations of target samples and controls between the groups. The results of expression of the investigated transcripts for each sample were tested for significance by a Two-sided Pair Wise Fixed Reallocation Randomisation Test^©^^47,48^. Difference in gene expression between control and treated samples with *p* < 0.05 were considered statistically significant.

## Supplementary Information

Supplementary Information.

## Data Availability

The datasets generated and/or analysed during the current study are available in the Zenodo repository, https://zenodo.org/record/7602272.
