# Supplementary material for: Spermidine dietary supplementation and polyamines level in reference to survival and lifespan of honey bees
Source: Sci Rep. 2023 Mar 15;13:4329. doi: 10.1038/s41598-023-31456-4 (PMC10017671; doi:10.1038/s41598-023-31456-4)
Supplement: Supplementary file 1 — Supplementary Information. [file 41598_2023_31456_MOESM1_ESM.pdf]

Supplement

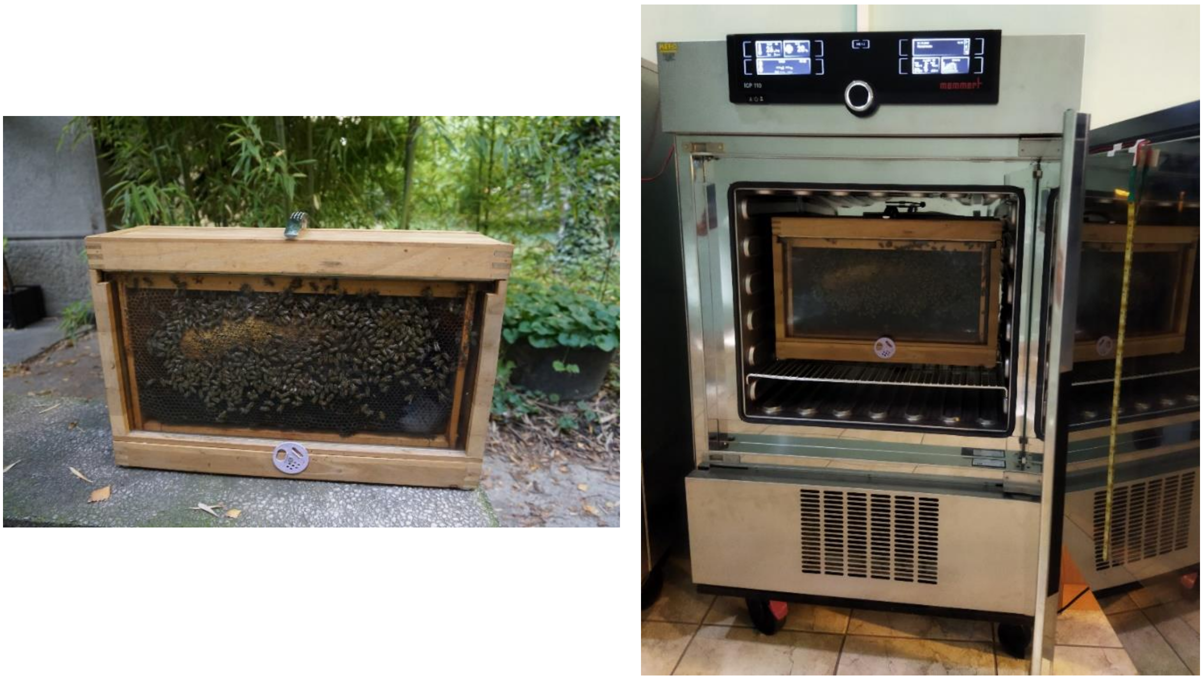

Supplement Figure 1. (A) Glass observation hive. (B) Glass observation hive in an incubator that imitates the conditions in hives.

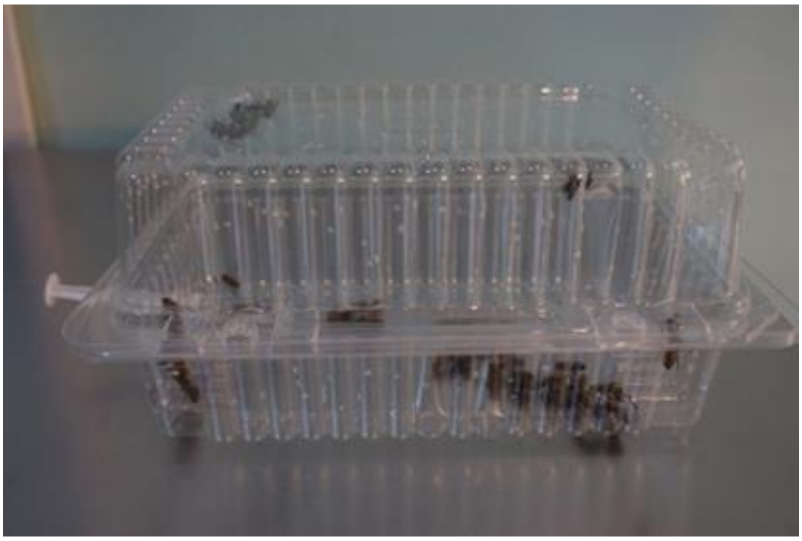

Supplement Figure 2. Experimental plastic box with a volume of 2L in which bees were raised in an incubator. There were between 30 and 55 bees in one box. Smooth aeration was provided by small holes in the box and the bees were fed using a plastic syringe that was pulled inside the box and fixed with adhesive tape.

Supplement Table 1. PCR primer sequences.

**Amplification target**		**Sequence**	**Efficiency**	**Reference**
Ribosomal protein 49 Rp49 (150 bp)	*Rp49.F*<br>*Rp49.R*	CGTCATATGTTGCCAACTGGT<br>TTGAGCACGTTCAACAATGG	88%	Lourenço rt al., 2008
Vitellogenin (137 bp)	*Vg.F*<br>*Vg.R*	TCAGTAACCAATGCGAGGGC<br>CGACATCTCGGTGTCCAATC	96%	Kim et al., 2022
Polyamine oxidase (125 bp)	*PAOX.F*<br>*PAOX.R*	AAGTGGAGCAGAAGTGGCAG<br>AGCCACGTGTGTATGGTTGT	93%	
Spermidine synthase (106 bp)	*SDS.F*<br>*SDS.R*	ACACCGAGCTTGACCAGATG<br>GCGAGTGACTTGTCCAATGC	106%	
Spermine oxidase (106 bp)	*SMOX.F*<br>*SMOX.R*	GTTTGCGCGTACAGAGTTCAT<br>TCGTGGCGATTTGGTATCGT	90%	
Ornithine decarboxylase (172 bp)	*ODC.F*<br>*ODC.R*	CAGTCTATTGGTGGGTGCGT<br>TACTCTGTAGGCGCTAGGCA	88%	
Spermine synthase (115 bp)	*SMS.F*<br>*SMS.R*	ACCCACATTTGACCCTAGTCG<br>ATGTACAGACGCCAGCGTAG	99%	
